# Determination of Acyclovir in Human Plasma Samples by HPLC Method with UV Detection: Application to Single-Dose Pharmacokinetic Study

**DOI:** 10.3889/oamjms.2015.011

**Published:** 2015-01-22

**Authors:** Dragica Zendelovska, Suzana Simeska, Emilija Atanasovska, Kalina Georgievska, Igor Kikerkov, Nikola Labachevski, Krume Jakovski, Trajan Balkanov

**Affiliations:** *Institute of Preclinical and Clinical Pharmacology and Toxicology, Medical Faculty, Ss. Cyril and Methodius University of Skopje, 50 Divizija 16, 1109 Skopje, Republic of Macedonia*

**Keywords:** acyclovir, HPLC, human plasma, pharmacokinetic study, Zovirax

## Abstract

**BACKGROUND:**

The aim of this study is estimation of pharmacokinetic parameters: C_max_, t_max_, t_1/2_, AUC_0-t_ and AUC_0-∞_ with the two-way analysis of variance, single observation (ANOVA) for two preparations containing acyclovir.

**OBJECTIVE:**

In order to evaluate pharmacokinetic study of acyclovir, method for quantitative determination of acyclovir in human plasma should be simple, rapid and reproducible. Therefore, the method is developed, validated and applied for analysis of acyclovir in plasma samples obtained from healthy volunteers.

**MATERIAL AND METHODS:**

High performance liquid chromatographic (HPLC) method with UV-detection for the determination of acyclovir in human plasma is presented. This method involves protein precipitation with 20 % (*V*/*V*) perchloric acid. The chromatographic separation was accomplished on a reversed phase C8 column with a mobile phase composed of 0.1 % (*V*/*V*) triethylamine in water (pH 2.5). No internal standard is required. UV detection was set at 255 nm. The method was successfully applied for the evaluation of pharmacokinetic profiles of acyclovir tablets in 24 healthy volunteers.

**RESULTS:**

The validation results shows that proposed method is rugged, precise (RSDs for intra- and inter-day precision ranged from 1.02 to 8.37 %) and accurate (relative errors are less than 6.66 %). The calibration curve was linear in the concentration range of 0.1-2.0 µg/ml and the limit of quantification was 0.1 µg/ml. The C_max_, t_max_ and AUCs for the two products were not statistically different (p>0.05), suggesting that the plasma profiles generated by Zovirax were comparable to those produced by acyclovir manufactured by Jaka 80 company.

**CONCLUSION:**

Good precision, accuracy, simplicity, sensitivity and shorter time of analysis of the method makes it particularly useful for processing of multiple samples in a limited period of time for pharmacokinetic study of acyclovir.

## Introduction

Acyclovir is considered the drug of choice for the treatment of initial and recurrent mucosal or cutaneous herpes simplex (HSV-1 and HSV-2) infections in immunocompromised adults and children, for the treatment of severe first episodes of genital herpes infections in immunocompetent patients; for the treatment of herpes simplex encephalitis, for the treatment of neonatal herpes infections and for the treatment of varicella-zoster infections in immunocompromised adults and children [1].

A number of assay methods have been reported for determination of acyclovir in biological fluids using capillary electrophoresis [2] or liquid chromatographic methods with pulsed amperometric detection [3], tandem mass spectrometry [4], fluorescence detection [5-7] or ultraviolet detection [8-15]. In the published methods, liquid-liquid extraction with acetonitrile or mixture of isopropyl alcohol and dichloromethane as solvent has been used for sample preparation [4, 8, 9]. The disadvantage of these methods employing liquid-liquid extraction (with grate chemical consumption) of acyclovir from biological fluids is that they involve several steps yielding poor separation from the serum endogenous interferences. In order to improve purification of biological samples Fernandez et al. proposed a solid-phase extraction method [10]. Solid-phase extraction technique is a fairly expensive procedure and suffers from sorbent drying between washing intervals which may result in cracking of the packing material. Sometimes, using liquid-liquid or solid-phase extraction there are problems in the dissolution of the residue after extraction and evaporation of organic layer under a gentle stream of nitrogen. All these methods are time consuming (usually up to 1 h) regarding to multiple steps of extraction, drying etc. Other authors proposed protein precipitation method for sample preparation with 20 % trichloroacetic acid or 7 % perchloric acid [5-7, 11-15]. Emami et al. reported that protein precipitation method can deteriorate the chromatographic column due to either high acidity or inadequate precipitation of protein contents in samples [8].

Therefore, we made some modifications in chromatographic conditions and have developed a HPLC method suitable for determination of acyclovir in plasma samples employing protein precipitation method for sample preparation. The advantage of the proposed method in comparison with other published papers is usage of 20 % perchloric acid for protein precipitation in order to achieve better separation of acyclovir peak from peaks of endogenous interferences compounds. This method enables simple and rapid isolation of acyclovir without using of internal standardization for the quantification and can increase the number of analysed samples in order to accomplish the pharmacokinetic study.

## Methods

### Materials

Acyclovir working standard was supplied by Recordati, Industria Chimica E Farmaceutica S.O.A., Italy. Triethylamine, o-phosphoric acid and perchloric acid were obtained from Merck (Germany).

### Instrument and chromatographic conditions

A series of parameters, including composition and pH of mobile phase, column packing, flow rate and detection wavelength, were tested with respect to the location and shape of the peak of acyclovir in the corresponding chromatograms. The final choice of the stationary phase giving satisfying resolution and run time was a reverse phase Hibar LiChrospher 100 RP8, 250 × 4.6 mm I.D. (5 μm, particle size), protected by a guard column LiChrospher RP8 4-4 mm (5 μm). A mobile phase consisting of 100 % water solution of 0.1 % (*V/V*) triethylamine with pH = 2.5 adjusted with concentrated ortho-phosphoric acid delivered by a pump Perkin Elmer LC series 200 was found to give best results. The mobile phase was filtered and degassed with helium. A flow rate was 1.2 ml/min. Chromatographic separations were performed at 25°C. An ultraviolet diode array detector (Perkin Elmer LC 235 C) was used for detection and 255 nm was chosen as optimal for determination of acyclovir. The samples were introduced in the column using an autosampler Perkin Elmer LC ISS Series 200 and the injection volume was 120 µl. The chromatographic system was controlled by the software package Turbochrom Version 4.1. plus and UV-spectrometric data were produced by TurboScan Version 2.0.

### Preparation of standards

Stock solution of 1000 µg/ml of acyclovir was prepared monthly in mixture of methanol and water in volume fractions 1:1 and stored at +4°C. No change in stability over the period of 1 month was observed. The working solutions were prepared by diluting appropriate portions of this solution with distilled water.

### Sample preparation

Human plasma samples were prepared by centrifuging (at 3000 rpm) of heparinized whole blood samples collected from healthy volunteers who later participated in a bioequivalence study of acyclovir.

Prepared plasma samples were stored at -20°C. Before the analysis blood plasma samples were thawed at 20°C for about 10 minutes. A 0.5 ml volume of the sample was transferred into a vial and treated with 0.1 ml solution of 20 % (V/V) perchloric acid. The mixture is vortexed for 20 s and centrifugated for 10 min at 10000 rpm. The supernatant was filtered using filter with pore size of 0.45 μm and 120 μl volume was injected into the HPLC system.

### Calibration curves

Typical calibration curves were constructed with six blank plasma samples spiked with appropriate amounts of the standard solutions. The calibration range was 0.1-2.0 µg/ml of acyclovir. The standard samples were prepared according to the procedure as unknown samples. The calibration curves were obtained by plotting the peak height versus concentration of acyclovir in µg/ml. The regression equations were calculated by the least-squares method.

### Application of the method

A total of 24 healthy male volunteers gave their written informed consent to participate in bioequivalence study of acyclovir. The health condition of the volunteers was established on the basis of anamnesis data, physical examination, ECG, biochemical and hematological analyses. This study was reviewed and approved by the Ethical Committee of the Faculty of Medicine, St. Cyril and Methodius University (Skopje, Macedonia). The study was a open, single dose, randomized, balanced, two-way crossover with a one-week wash-out period. Two weeks before investigation volunteers did not receive any drugs. The subjects were administered a single 400 mg oral dose of acyclovir.

Plasma samples were obtained before the administration of the drug (0 time) and at 12 time points following a single 400 mg oral dose of acyclovir. Following collection, the samples were stored at -20 ºC. After thawing, 0.5 ml plasma was analyzed as described before.

## Results

### Method validation

**Figure 1:**
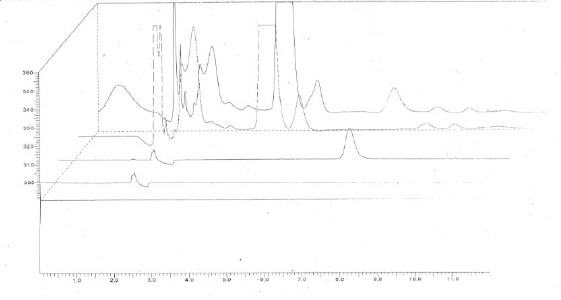
*Chromatograms of diluents (a), standard solution of acyclovir (b), blank plasma sample (c) and spiked plasma sample with acyclovir (d)*.

Under the chromatographic conditions described, acyclovir peak was well resolved. Endogenous plasma components did not give any interfering peaks. A typical chromatogram of diluent (a), standard solution of acyclovir (b) produced by the developed HPLC method is shown in Fig. 1. Retention time of acyclovir is 7.75 min. Chromatograms of blank plasma in comparison to spiked samples prepared using the method of standard addition are shown in Fig. 1 (c, d).

Linearity of the method was tested in three different days at six concentration points ranged from 0.1 to 2.0 µg/ml of acyclovir in plasma samples. Respective regression equation was: y = 9763.4·γ - 24.321. The correlation coefficient was 0.9985.

In one day and in 3 different days, spiked samples from each concentration used for construction of calibration curves were prepared in triplicate and analyzed by the proposed HPLC method. Then, the corresponding coefficients of variation were calculated. The intra- and inter-day variations of the method throughout the linear range of concentrations are shown in Table 1.

**Table 1:** Intra-and inter-day precision and accuracy data.

Acyclovir nominal concentration (μgml^−1^)	Intra-day		Inter-day	
	
	Mean (n=3) observed concentration (μgml^−1^)	Relative standard deviation (%)	Mean (n=9) observed concentration (μgml^−1^)	Relative standard deviation (%)
		Precision		

0.1	0.10	1.15	0.09	2.72
0.2	0.21	4.62	0.21	4.46
0.5	0.52	1.02	0.51	1.74
0.9	0.89	3.19	0.87	3.11
1.5	1.43	2.58	1.41	3.52
2.0	2.08	8.37	1.99	6.88

Accuracy		Relative error (%)		Relative error (%)

0.3	0.31	3.33	0.31	3.33
0.7	0.74	5.71	0.68	-2.86
1.2	1.20	0.0	1.12	-6.66

Intra- and inter-day accuracy was determined by measuring plasma quality control samples at low, middle and high concentration levels. An indication of accuracy was based on the calculation of the relative error of the mean observed concentration as compared to the nominal concentration. Accuracy data are presented in Table 1.

The limit of quantification was defined as the lowest amount detectable with a precision of less than 15 % (n=5) and an accuracy of ±15 % (n = 5). The limit of quantification was found to be 0.1 µg/ml for plasma samples.

Stability of acyclovir in plasma samples was investigated using spiked samples at two different concentration levels prepared in duplicate. Spiked samples were analysed after different storage conditions: immediately, after staying in an autosampler for 2, 12 and 24 hours, after one and two freeze/thaw cycles and after 1 month stored at –20°C. The results from this investigation show that acyclovir added to plasma samples is stable in the different storage conditions (relative errors were ranged from 1.15 % to 6.98 %).

Ruggedness was performed on the second column of the same type by determining repeatability and accuracy analysing three series of quality control samples. From three determinations per concentration relative standard deviations were ranged from 0.58 % to 4.49 % and relative errors were ranged from -1.57 % to 4.0 % which means that this HPLC method for determination of acyclovir in spiked human plasma samples is rugged.

**Figure 2:**
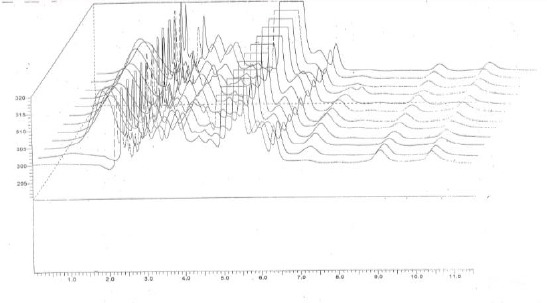
*Chromatograms of plasma samples from healthy volunteers dosed with 400 mg acyclovir 0.33 h, 0.66 h, 1 h, 1.33 h, 1.66 h, 2 h, 3 h, 4 h, 6 h, 8 h, 12 h and 24 h post dose respectively (from the first point sampling 0.33 h followed by the rest point samplings)*.

On the other hand, the method in this report has sufficient sensitivity and reproducibility to permit the pharmacokinetic studies. The developed HPLC method was used for analysis of plasma samples from healthy volunteers after oral administration of 400 mg acyclovir. Typical chromatograms of plasma extracts of a volunteer after administration of 400 mg acyclovir are shown in Fig. 2. Chromatograms showed no interfering peak at the acyclovir peak position.

#### Pharmacokinetic study of acyclovir

**Figure 3:**
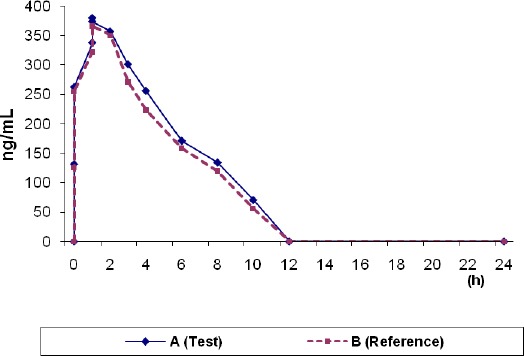
*Plasma concentration – time curve of Acyclovir*.

The described HPLC method was successfully applied in a bioequivalence study of acyclovir formulation on 24 healthy volunteers. Fig. 3 presents the mean plasma acyclovir concentration-time profiles after oral administration of 2x200 mg test acyclovir formulation or 2x200 mg acyclovir (a reference formulation of acyclovir - Zovirax). As can be seen from Fig. 3, the character of both curves is practically the same. Following oral administration of acyclovir and Zovirax tablets, maximum plasma concentration of 452 and 432 ng/ml were achieved after 1.39 h and 1.40 h, respectively.

The corresponding pharmacokinetic parameters (mean ± SD) obtained by 24 independed analyses and the relative bioavailability of the generic acyclovir tablet are presented in Table 2.

**Table 2:** Mean pharmacokinetic parameters of acyclovir after a single 400 mg oral dose

Pharmacokinetic parameter	Acyclovir tablet	Zovirax tablet
C_max_ (ng ml^-1^)	452 ± 110	432 ± 108
t_max_ (h)	1.39 ± 0.55	1.40 ± 0.38
t_1/2_ (h)	3.59 ± 1.1	3.87 ± 1.54
AUC_0-t_ (ng h ml^-1^)	1782 ± 600	1687 ± 627
AUC_0-∞_ (ng h ml^-1^)	2439 ± 623	2485 ± 955
F_r_	100.6 ± 5.5	-

C_max_ – mean plasma acyclovir concentration; t_max_ – time to achieve the peak concentration t_1/2_ – elimination half-life; AUC_0-t_ – area under the plasma concentration-time curve from 0 to t; AUC_0-∞_ - area under the plasma concentration-time curve from 0 to infinity; F_r_ – relative bioavailability defined as AUC_acyclovir_/AUC_Zovirax_

### Discussion

In our laboratory a series of studies were conducted in order to develop a convenient and easy-to-use method for quantitative analysis of acyclovir in human plasma. Several HPLC method variables with respect to their effect on the separation of acyclovir from the matrix were investigated.

In our extensive preliminary experiments a series of aqueous mobile phases containing buffer solutions with different pH values in combination with different modifiers including acetonitrile, methanol and triethylamine with different volume fractions were tested. The results were most satisfactory when mobile phase consisted of 100 % water solution of 0.1 % (*V/V*) triethylamine with pH=2.5.

A set of column packing including C8 and C18 with different lengths and particle sizes were tested and the LHibar LiChrospher 100 RP 8 packing showed best separation. Among several flow-rates tested (0.8-2 ml/min) the rate of 1.2 ml/min was the best with respect to location and resolution of the peaks of acyclovir from the interfering peaks. The elution was monitored in the whole UV region and the wavelength of 255 nm exhibited the best detection.

Results of method validation for intra-day precision show that RSDs ranged from 1.02 to 8.37 % and for inter-day precision, RSDs ranged from 1.74 to 6.88 %. These data indicate a considerable degree of precision and reproducibility for the method both during one analytical run and between different runs.

On the other hand, the values for relative errors at all three concentrations studied for plasma samples are less than 6.66 % and it is obvious that the method is remarkably accurate which ensures obtaining of reliable results.

The present method was applied in bioequivalence study of two preparations containing acyclovir. The statistical analysis of pharmacokinetic parameters: C_max_, t_max_, t_1/2_, AUC_0-t_ and AUC_0-∞_ with the two-way analysis of variance, single observation (ANOVA) shows that there are no differences between the compared drugs. The relative bioavailability of the investigated formulation, i.e., the acyclovir tablet (manufactured by Jaka 80 Company) was 100.6 % of that obtained for the proprietary product, Zovirax. Based on these results, it may be concluded that acyclovir tablets are bioequivalent to Zovirax tablets.

In conclusion, the developed HPLC method employing protein precipitation is simple and convenient for the determination of acyclovir in plasma samples. Acyclovir has been successfully separated. The proposed method is simply, rapid and provides efficient clean up of the complex biological matrix. The validation data demonstrate good precision and accuracy, which proves the reliability of the proposed method. Finally, the method has been implemented to pharmacokinetic study of acyclovir.
